# Antibacterial Electrodeposited Copper-Doped Calcium Phosphate Coatings for Dental Implants

**DOI:** 10.3390/jfb14010020

**Published:** 2022-12-29

**Authors:** Camille Pierre, Ghislaine Bertrand, Iltaf Pavy, Olivier Benhamou, Christian Rey, Christine Roques, Christèle Combes

**Affiliations:** 1 CIRIMAT, Université de Toulouse, CNRS, Toulouse INP-ENSIACET, 31030 Toulouse, France; 2 Laboratoire de Génie Chimique, Université de Toulouse, CNRS, Université Paul Sabatier, Faculté des Sciences Pharmaceutiques, 31062 Toulouse, France; 3 Arts Loi Dental Clinic, Rue de la Loi 28, 1040 Bruxelles, Belgium

**Keywords:** dental implants, calcium phosphate coating, copper, antibacterial, biofilm model, peri-implantitis

## Abstract

Dental implants provide a good solution for the replacement of tooth roots. However, the full restoration of tooth functions relies on the bone-healing period before positioning the abutment and the crown on the implant, with the associated risk of post-operative infection. This study aimed at developing a homogeneous and adherent thin calcium phosphate antibacterial coating on titanium dental implants by electrodeposition to favor both implant osseointegration and to limit peri-implantitis. By combining global (XRD, FTIR-ATR, elemental titration) and local (SEM, Raman spectroscopy on the coating surface and thickness) characterization techniques, we determined the effect of electrodeposition time on the characteristics and phases content of the coating and the associated mechanism of its formation. The 1-min-electrodeposited CaP coating (thickness: 2 ± 1 μm) was mainly composed of nano-needles of octacalcium phosphate. We demonstrated its mechanical stability after screwing and unscrewing the dental implant in an artificial jawbone. Then, we showed that we can reach a high copper incorporation rate (up to a 27% Cu/(Cu+Ca) molar ratio) in this CaP coating by using an ionic exchange post-treatment with copper nitrate solution at different concentrations. The biological properties (antibiofilm activity and cytotoxicity) were tested in vitro using a model of mixed bacteria biofilm mimicking peri-implantitis and the EN 10993-5 standard (direct contact), respectively. An efficient copper-doping dose was determined, providing an antibiofilm property to the coating without cytotoxic side effects. By combining the electrodeposition and copper ionic exchange processes, we can develop an antibiofilm calcium phosphate coating on dental implants with a tunable thickness and phases content.

## 1. Introduction

Dental implants provide a good solution for the replacement of tooth roots. Indeed, they restore the ability to eat and speak while preserving jawbone density. The proper design of a dental implant material is aimed at providing the requisite durability, functionality, and biological response in long-term use. Nevertheless, two major drawbacks still exist. The first is the long bone-healing time (3–6 months) that elapses between the implantation and the positioning of the abutment and the crown on the dental implant, which fully re-establish the function of the tooth. The second corresponds to post-operative infections that, when occurring, can drastically reduce the lifespan of the implant.

Post-operative infections and especially peri-implantitis affect soft and hard tissues leading to bone loss around the implant and can cause implant loosening. Peri-implantitis occurs in 5 to 10% of implantation surgeries [1,2] and becomes a major concern among 28–56% of patients with the risk of implant loss [3,4]. It has been described as a destructive inflammatory lesion associated with surgical complications resulting in bone loss (loss of osseointegration), peri-implant pocketing, and finally, implant failure [2,3]. Additional pain and anxiety are the main consequences of such problems, as well as increased costs in the event of a second surgery [5,6]. Effective prevention and management of peri-implantitis are essential in maintaining the quality of life and health of the patients [7] and reducing the financial burden of the healthcare system. The progressive invasion of pathogenic bacteria leads to their adhesion to the implant surface and the junction with soft tissue, and the structuration and growth of the microbiota under the biofilm [8,9]. This could induce a persistent inflammatory response in the gingiva and its surrounding combined alveolar bone in response to secreted endotoxins and, ultimately, to alveolar bone loss, which is peri-implantitis [10]. The pathogenesis and clinical symptoms of peri-implantitis are close to that of periodontitis [11], and several studies have reported common microbiota [12,13] with the initial (*streptococci*, *actinomycetes*) and later colonizers (especially Gram-negative anaerobes). Rarely, opportunistic micro-organisms such as enteric rods and *Staphylococcus aureus* were isolated [14]. Recent studies using culture-independent methods revealed the microbial specificity regarding the active sites and diversity of this disease [15,16,17]. The bacteria generally present during peri-implantitis are anaerobic Gram-negative bacteria such as *Porphyromonas gingivalis*, *Prevotella intermedia*, *Aggregatibacter actinomycetemcomitans*, *Tannerellas forsythias*, *Treponema denticola*, *Fusobacterium* sp., *Campylobacter* sp., *Prevotella nigrescens*, and *Campylobacter* [2,15,18]. The main pathogens of peri-implantitis were thus Gram-negative bacteria, especially *P. gingivalis* [19], and the average colony-forming units of bacteria in peri-implantitis sites were higher than that in healthy sites, underlying an increase within the biofilm which becomes more pathogenic [15]. The treatment of peri-implantitis includes non-surgical and surgical approaches with no certainty of effectiveness, and no standard of care treatment is still defined [20,21,22,23]. Therefore, it seems important to first evaluate the limitation of bacterial adhesion and growth on the implant surface to prevent biofilm formation.

Titanium and its alloys are widely used as implants in the dental field [24] with surface modifications to improve surface roughness and osseointegration. New implant coatings are characterized by a combination of functions including antimicrobial/antibiofilm ones. For this purpose, antibiotics have been widely used [25,26,27,28].

Nevertheless, the main disadvantage of antibiotics is related to the increasing development of bacterial strains resistant to antibiotics and the narrow spectrum of activity which complicate the treatment of infections caused by several bacteria [5,6,29]. Another alternative is the use of non-antibiotic organic antimicrobial agents such as chlorhexidine, chloroxylenol, and polyhexamethylene biguanide, but some studies have shown cellular damage [30]. Finally, the use of bioactive materials and inorganic antimicrobial agents such as ions/elements is attractive because they can show a high antibacterial activity with a large spectrum, a good biocompatibility depending on their concentration, and a good stability [6,29,31]. Even if silver is the most known [32,33], it is also possible to use copper, zinc, or fluorine [26,30].

To deliver antibiotic agents as ions/elements, the most appropriate strategy consists of depositing a calcium phosphate (CaP) coating on the titanium dental implant to use it as a material platform that can be doped with the active ions/elements. For this purpose, several processes have been proposed: plasma spraying, pulsed laser deposition, electrophoretic deposition, soaking process, and electrochemical deposition [34,35,36]. Among them, electrodeposition, that is, a wet and low-temperature process, offers major advantages [37]. Indeed, it allows the treatment of small pieces with complex shapes such as dental implants. Moreover, the thickness, the composition, and the microstructure of the coating can be modulated by adjusting the experimental conditions (pH, temperature, ions concentrations, etc.) [38]. It has especially been proven that non-stoichiometric nanocrystalline apatite, similar to the natural bone mineral, involving highly mobile ions that provide an exceptional surface reactivity, can be electrodeposited on metallic surfaces [39].

Copper [40] and zinc [41] have already been introduced in CaP-electrodeposited coating, and have shown an antibacterial effect [42,43,44]. The doping metal is usually introduced directly in the electrolyte and the antibacterial properties of the doped CaP coating are evaluated on one or separated bacteria strains. From the literature, it appears that no specific multispecies biofilm model mimicking peri-implantitis has been implemented to assess the doped CaP coating on dental implants. In addition, a two-step coating process including CaP electrodeposition and then a doping step with a controlled doping cation concentration could be a versatile route to customize antibacterial dental implants.

The purpose of this study was to develop a homogeneous and adherent thin (a few micrometers) CaP antibacterial coating on titanium dental implants by electrodeposition to favor implant osseointegration and limit the postoperative peri-implantitis risks. After studying the effect of electrodeposition time on the characteristics of the coating and the associated mechanism of formation, its doping with copper ions was investigated using an aqueous post-treatment in order to provide an antibacterial property to the coating without being cytotoxic. In vitro microbiological tests were carried out to demonstrate the preventive antibacterial activity of such a Cu-doped CaP coating using a model of mixed biofilm adapted from Guggenheim et al. [45], combining early colonizers and the main Gram-negative anaerobic and facultative anaerobic bacteria considered as pathogens in peri-implantitis.

## 2. Materials and Methods

### 2.1. Sample Preparation

All experiments were performed on cylindrical titanium samples of 6 mm length and 6 mm diameter with a flattened surface for characterization purposes. Optimized coatings were also deposited on titanium dental implants. All the substrates were made of commercially pure grade 4 titanium (cpTi). The machined samples were successively sandblasted with alumina particles (F100) and acid-etched with a solution including H_2_SO_4_:HCl:H_2_O in 3:1:1 (vol %) proportion at 40 °C for 20 min according to an already published protocol [36].

### 2.2. Electrodeposition of Calcium Phosphate Coating

The experimental setup is composed of three electrodes: the sample to be coated is placed at the cathode, a platinum counter electrode, and a saturated calomel electrode (SCE) as reference. The experiments were carried out using a potentiostat/galvanostat *SI 1287* (Solartron Analytical, Farnborough Hampshire, UK) controlled by *CorrWare^®^* software.

The electrolyte solution was prepared using Ca(NO_3_)_2_·4H_2_O and NH_4_H_2_PO_4_ as calcium and phosphate sources respectively to obtain concentrations of [Ca] = 0.042 M and [P] = 0.025 M corresponding to a Ca/P ratio of 1.67. The temperature of the electrolytic solution was set at 60 °C and the stirring at 200 rpm. Figure S1 shows the experimental set.

Electrodeposition was realized with a constant potential of −1.6 V/SCE for a variable duration from 30 s to 10 min. −1.6 V/ESC was chosen for all the experiments, as we found, from a preliminary study (data not shown), that this constant potential showed a good compromise in terms of electrodeposition time/thickness of the coating, and more importantly, on the homogeneity of the titanium substrate covering.

### 2.3. Ionic Exchange

The association of copper ions with the as-electrodeposited calcium phosphate coating was realized by an ionic exchange post-treatment on CaP-coated samples obtained after 1 min of electrodeposition. It consists of immersing a CaP-coated sample during 15 min in an aqueous bath at different concentrations of copper (0.001; 0.005; 0.01; 0.05; 0.1; or 0.15 M of Cu(NO_3_)_2_·2.5H_2_O salt (ACS, 98.0–102.0 %, Alfa Aesar)) and then rinsing it with deionized water and drying it at room temperature.

### 2.4. Coating Characterization

After the coating process, sample surface was observed by SEM (LEO 435VP, ZEISS, Oberkochen, Germany). Micrographs were recorded in secondary electrons mode at a voltage of 15 KeV and a probe intensity of 150 pA to determine the surface microstructure and the coverage of titanium substrate with the CaP coating. Moreover, cross-sections were examined to allow coating thickness measurements. An average and a standard deviation were calculated from ten measurements carried out on three coated samples obtained using the same electrodeposition conditions.

The elemental composition analysis of the coating, before and after doping, was investigated by energy dispersive X-ray spectroscopy (EDX). A voltage of 15 KeV, a probe intensity of 1500 pA, and an acquisition time of 100 s were set.

The physico-chemical characterization of the coating permitted us to determine the phase(s) of calcium phosphate constituting the coating. With the experimental conditions (pH and T) used during the electrodeposition of CaP coating and according to the literature, brushite (DCPD), octacalcium phosphate (OCP), non-stoichiometric apatite analogous to bone mineral (ns-HAP), or hydroxyapatite (HAP) are the CaP phases that could potentially precipitate on the substrate [38,46,47]. Complementary techniques, XRD as well as FTIR and Raman spectroscopy, were implemented to further characterize the coating.

The calcium phosphate phase(s) of the coatings were investigated using an X-ray diffractometer (D8-Advance, BRUKER, Billerica, MA, USA) in grazing incidence mode (2°) to permit the analysis of the coating directly on the substrate. The XRD data were collected using a Cu anticathode (wavelength = 1.54184 Å), from 2Θ = 3° to 80° with a step of 0.03° and a counting time of 6 s.

FTIR spectroscopy analysis (FTIR spectrometer iS50, Nicolet, Thermo Scientific, Thermo Electron Scientific Instruments LLC, Madison, WI, USA)) was performed in the middle infrared range and attenuated total reflectance (ATR) mode using a diamond crystal in order to analyze the CaP coatings directly on the substrate. Several points were analyzed to check the chemical composition homogeneity of the coating.

Raman spectroscopy combined with a confocal microscope (LabRAM HR 800, HORIBA Jobin Yvon, Longjumeau, France) allowed for a focus on a special area. A 532 nm laser source, a 1800 grooves/mm grating, and a ×100 microscope objective were used for the acquisitions.

DCPD, OCP, ns-HAP, and HAP reference compounds were synthesized at the CIRIMAT Laboratory using previously published protocols and their purity was checked by XRD by comparing the obtained diffractograms with JCPDS files n° 01-072-0713, n° 0-026-1056, n° 00-046-0905, and n° 09-0432, respectively.

Coating thickness was measured by SEM observation as explained before and also determined by two other complementary techniques: calotest technique (CSEM Instrument) and atomic force microscopy (AFM, STM 5500, SCIENTEC). The AFM measurements of the height of a step created on the titanium surface between a polished part of this substrate and a coated one were made in tapping mode with a frequency of 300 kHz. A silicon tip having a spring constant of 25 N/m, a nominal radius of curvature of 4 nm, and scans of 60 µm were used.

The mechanical stability of the coating on the dental implant (obtained after 1 min of electrodeposition) was checked using a screwing/unscrewing test mimicking an implantation procedure in an artificial jawbone, as already published [36]. After the test, the dental implant was cleaned of artificial jawbone particles with compressed air and the implant surface was observed by SEM.

Regarding the doped coating, calcium and copper concentrations were determined by atomic absorption spectroscopy (AAS, Contr AA 300–ANALYTIK JENA). A mixture of nitrogen protoxide and acetylene was used for the flame for calcium titration whereas a blend of air and acetylene was selected for the copper one. Before analysis, each coating was dissolved in nitric acid solution (0.06 M). Moreover, for calcium titration, matrix modifiers, CsCl and La(NO_3_)_3_, with a concentration of 2 g/L each were added to the solution to favor the analysis by limiting the interference due to phosphate ions in the solution. The amount of copper ions incorporated in the coating was calculated as follows with regard to the total cations in the coating:

$$\%\;\mathrm{Cu}\;\left(\mathrm{mol}\right)=\left(\frac{\left[\mathrm{Cu}\right]}{\left[\mathrm{Cu}\right]+[\mathrm{Ca}]}\right)\;\times \;100$$

Inductively coupled plasma spectrometry (ICP, ICP Ultima Expert, HORIBA) was used to measure calcium and phosphorus within the same sample and the Ca/P ratio corresponding to the coating was calculated. For this purpose, the coating was dissolved as described previously for the copper analysis by AAS. Analyses were performed in triplicate.

### 2.5. In Vitro Biological Tests

#### 2.5.1. Coupons under Assay

Non-doped CaP-coated titanium coupons and copper-doped CaP-coated titanium coupons (Cu-doped samples after exchange in 0.001, 0.005, or 0.01 M copper ion solutions) were gamma-sterilized (gamma radiation dose of 25–40 kGy) and used for evaluation of biofilm formation and cytotoxicity on Vero cells.

#### 2.5.2. Tested Bacteria

Reference strains were obtained from Institut Pasteur Collection (Paris, France) and Table 1 shows a list of bacterial strains from primary colonizers to the largely anaerobic secondary colonizers, culture media, and incubation conditions used.

#### 2.5.3. Activity on Biofilms

Biofilms were established according to the Zürich biofilm model [45] considering pioneer and colonizer species.

Saliva samples were obtained from healthy donors after giving informed consent and collected on salivette^®^ then immediately filtered on 0.45 μm membranes before pooling. Before each experiment, titanium coupons were put into wells of a 24-well sterile microplate. Titanium coupons were first covered with the pooled saliva for 1 h or 15 min [45,48,49] at ambient temperature. After saliva removal, carriers were covered with 1.6 mL of 50% saliva-50% modified universal fluid medium (mFUM) (67 mmol of Sørensen buffer/L, final pH = 7.2) [50]. Wells were then inoculated with mixed bacterial suspensions (200 μL) prepared with equal volumes of each species suspension freshly prepared in Tryptone salt (BioMérieux) at defined concentrations, as follows:
*S. gordonii*CIP 105258T10^3^/mL*A. naeslundii*CIP 103128T10^5^/mL*P. micra*CIP 105294T10^6^/mL*F. nucleatum*CIP 101130T10^7^/mL*A. actinomycetemcomitans*CIP 52106T10^7^/mL*P. intermedia*CIP 10360710^7^/mL*P. gingivalis*CIP 10368310^7^/mL

Then, microplates were incubated anaerobically at 36 ± 1 °C.

Numeration of adherent cultivable bacteria was performed after 4 h and 24 h from inoculation in an attempt to differentiate both anti-adhesion and anti-proliferation effects. Biofilms were harvested by scraping with a sterile blade in physiological saline solution (5 mL). Bacterial suspensions were homogenized and serially diluted (10 fold). One hundred microlitres of each dilution were then plated onto Wilkins–Chalgren agar supplemented with 5% horse blood cells, and onto Wilkins–Chalgren agar supplemented with 5% horse blood cells and with 0.075 g/L of vancomycin (selective agar for Gram-). Plates were incubated anaerobically at 36 ± 1 °C for 5 to 7 days, then for a 14-day supplementary incubation for *P. gingivalis* count, and the number of colony-forming units (CFUs) was determined for the total flora, Gram-positive and Gram-negative bacteria, considering the macroscopic and microscopic morphologies. This protocol was set first on sandblasted, and acid-etched machined titanium samples (see in Section 2.1, data not shown), and then applied to CaP-coated samples. Each concentration was tested independently and in duplicate (control versus loaded samples). For each assay and each condition, CFU counts were expressed in log, then mean ± SD were calculated.

### 2.6. Scanning Electron Microscopy (SEM)

Prior to observations, the samples were fixed in 2.5% glutaraldehyde at 4 °C for 1 h and dehydrated in a graded series of aqueous ethanol solutions (30, 50, 80, and 99% of ethanol). Finally, samples were sputtered with gold-palladium and examined in a Quanta 250 FEG (Field Emission Gun) FEI microscope (CMEAB: Centre de Microscopie Electronique Appliquée à la Biologie–Faculté de Médecine–Toulouse, France).

### 2.7. Statistical Analysis

For antibiofilm activity evaluation, assays were performed twice. Total CFUs were log-transformed to fit a normal distribution and results were expressed as mean values ± standard deviation (SD). Statistical analysis was performed with the *t*-test for intergroup comparison. Statistical significance was set at the *p* < 0.05 level.

## 3. Results

### 3.1. Influence of Electrodeposition Time

The electrodeposition parameters (i.e., [Ca], [P], applied potential, stirring, pH, temperature) were fixed as detailed in the experimental part and the time of electrodeposition was studied: 30 s, 1, 2, 5, or 10 min. As expected, it appears that the longer the electrodeposition time, the more the implant is visually evolving from metallic to a white color, which could indicate a thicker coating. In addition, after a long electrodeposition time (5 and 10 min), some metallic surfaces of the substrate could be observed, probably due to coating delamination.

**Figure 1:**
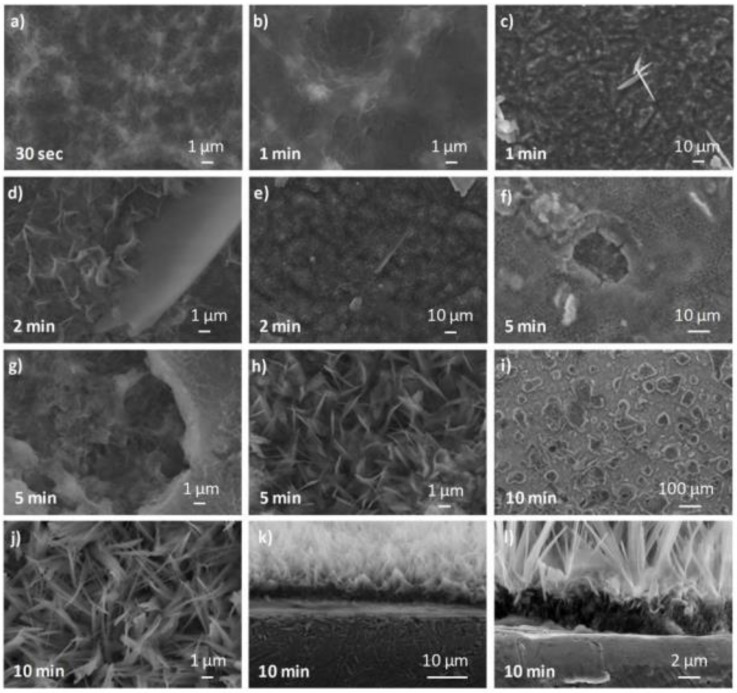
SEM micrographs of calcium phosphate coatings obtained after an electrodeposition time of: (**a**) 30 s; (**b**,**c**) 1 min; (**d**,**e**) 2 min; (**f**–**h**) 5 min; and **(i**–**l**) 10 min; (**k,l**) Cross-section of the substrate-coating interface for the 10 min electrodeposited sample.

SEM micrographs of the different coatings show various morphologies. Indeed, for an electrodeposition time of 30 s, a layer of nanometric needles covering the entire substrate surface can be observed (Figure 1a). After 1 min, this layer is still visible (Figure 1b) and a few micrometric thin platelet crystals of 10–20 µm appear on this layer (Figure 1c). After 2 min, the nano-needles and the micro-platelets seem larger (Figure 1d,e). Then, craters of about 10–30 μm are observed (Figure 1f) after 5 min of electrodeposition, probably due to H_2_ bubbles created by the reduction of water on the cathodic sample surface. Different morphologies can be observed on this coating (t = 5 min): nano-needles and micro-platelets similar to those observed for short electrodeposition times (Figure 1g), and micrometric needles (Figure 1h). The same observation can be done for a coating formed after 10 min of electrodeposition where craters are also visible, leading to an inhomogeneous surface coverage (Figure 1i). We can note that the micrometric needles (of about 4–8 µm long) seem longer and thinner (Figure 1j) compared to those observed for 5 min of electrodeposition (Figure 1h). We may assume that the micrometric needles observed for the longer electrodeposition time originate from the nanometric ones visible for the shorter electrodeposition time. To confirm this hypothesis, a cross-section of the coating formed after 10 min of electrodeposition was observed (Figure 1j,k). It appears that the coating is composed of two layers. The first one, close to the substrate (thickness of about 2–3 μm, Figure 1l), is composed of nanometric needles randomly oriented similar to those observed for the short time of electrodeposition. The second one (thickness of about 10 μm, Figure 1l) is formed by micrometric needles oriented perpendicularly to the substrate surface as observed for 5 and 10 min of electrodeposition, showing an oriented CaP crystal growth.

Regarding the choice of electrodeposition time for this study, it was observed that the coatings obtained from a long duration (5 and 10 min) are very heterogeneous because of the craters created by the dihydrogen bubbles that emerge from the surface of the sample during electrodeposition (see Equations (2) and (3) in Section 4.1 for further details). It could lead to a non/less-cohesive coating. In order to choose the optimum electrodeposition time, the coating thickness was measured to assess the coatings that are compliant with the specification of less than 2 µm. The coating thickness was estimated using SEM observation of the cross-sections, calotest for thick coatings, and AFM step-height measurements for the thinnest ones (limited to 3.6 µm thick). Table 2 reports the thicknesses evaluated for all the deposition times.

The differences between the values measured with the three techniques may be attributable to the morphology of the various phases coexisting in the coating: layer of nano-needles rather well-aligned versus large platelets emerging with high angles outside the needle layer. Coatings electrodeposited in less than 2 min (thickness ≤ 2 μm) can be selected for the biological study.

The different morphologies observed previously by SEM (nano-needles, micro-needles, and platelets) could be related to distinct calcium phosphate phases. That is why the calcium phosphate phases formed during electrodeposition were studied using XRD as well as FTIR and Raman spectroscopy.

**Figure 2:**
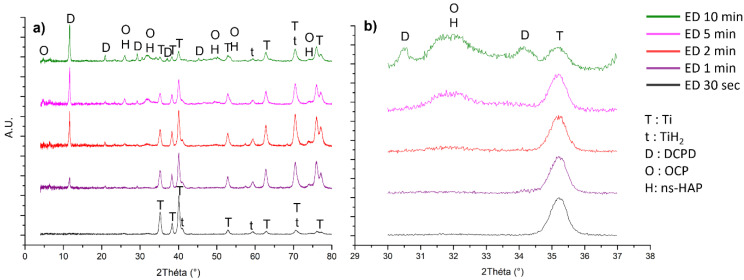
XRD patterns of the calcium-phosphate-coated samples obtained after different electrodeposition times (30 s, 1, 2, 5, and 10 min): (**a**) from 2θ = 4° to 80°, and (**b**) from 2θ = 29° to 38°.

The XRD patterns of the three reference calcium phosphate compounds (DCPD, OCP, and ns-HAP) as well as that of the coated samples for 30 s, 1, 2, 5, and 10 min of electrodeposition are shown in Figure 2.

For all of these, peaks identified at 35, 38.5, 40, 53, 63, 71.5, and 72.5° (Figure 2a) correspond to the titanium constituting the substrate according to the JCPDS file n°00-005-0682. The ones at 41 and 59.5° are attributed to TiH_2_ (JCPDS file n°00-009-0371) due to the acid-etching reaction of titanium with sulphuric acid already observed in previous works [45,47,51]. Coatings obtained after 1, 2, 5, and 10 min of electrodeposition show a peak at 11.5° indicating that DCPD takes part in these coatings. This is confirmed by the peaks at 21, 29, 30.5, 34, 36, and 45°, according to the JCPDS file n°09-0077 of the DCPD reference. Moreover, a broad peak between 31 and 33° can be observed from an electrodeposition time of 2 min (Figure 2b) which could correspond to OCP or ns-HAP, according to the JCPDS files n°00-026-1056 and n°00-046-0905, respectively. This can be confirmed by the peak at 26°, the low-intensity ones at 47 and 50°, as well as the shoulders at 54 and 73°. Indeed, it is difficult to distinguish these two calcium phosphate phases because most of their characteristic peaks are at similar or very close positions on the XRD patterns. However, a peak at 4.5° attributable to the OCP phase could allow us to differentiate these two CaP phases; the latter can be observed on the XRD patterns for the coating obtained after 5 and 10 min of electrodeposition. Nevertheless, it is important to note that a strong diffuse background at low diffraction angles is observed and thus corrected in Figure 2. Therefore, the peak observed around 4.5° could also be an artefact related to the signal treatment. Moreover, the XRD patterns present a low resolution, probably due to the low crystallinity of the CaP phases in the coatings. It is also important to note that intensities of the peaks corresponding to the CaP phases increase with the electrodeposition time compared to those corresponding to the substrate, which decrease. This phenomenon could be related to the average coating thickness which increases with the electrodeposition duration (samples with white surface).

Finally, none of the peaks corresponding to the calcium phosphate phases can be observed on the XRD pattern of the sample coated during 30 s of electrodeposition, which does not allow any conclusion to be drawn on the phases constituting this coating. This could be due to the very low thickness of the coating for such a short electrodeposition duration (≤1.6 μm; see Table 2). Moreover, the substrate roughness (1.4 < Ra < 1.6 µm) makes the XRD characterization complicated. Indeed, characterizing a very thin coating on a rough substrate is challenging.

The ATR-FTIR spectroscopy analysis of DCPD, OCP, ns-HAP, and the coated samples after 1 and 10 min of electrodeposition (Figure S2) confirmed the XRD results, especially for the 10 min coated sample. The analysis of the spectrum of the 1 min-coated sample is more complicated due to the poor resolved bands explained by a lower crystallinity of the phase(s) constituting this coating, by its lower coating thickness, and also, by the substrate roughness (1.4 < Ra < 1.6 µm), making the direct contact between the diamond crystal of the ATR device and the coating difficult.

Therefore, based on the XRD and FTIR-ATR spectroscopy global analyses, it is possible to conclude that the different coatings, except the one formed after 30 s of electrodeposition, are composed of DCPD as well as OCP and/or ns-HAP. We then investigated the electrodeposited coatings by using a local spectroscopic analysis technique, Raman microspectroscopy, to further examine the coating composition. The Raman and FTIR spectroscopy band assignments were performed according to Rey et al. [52].

**Figure 3:**
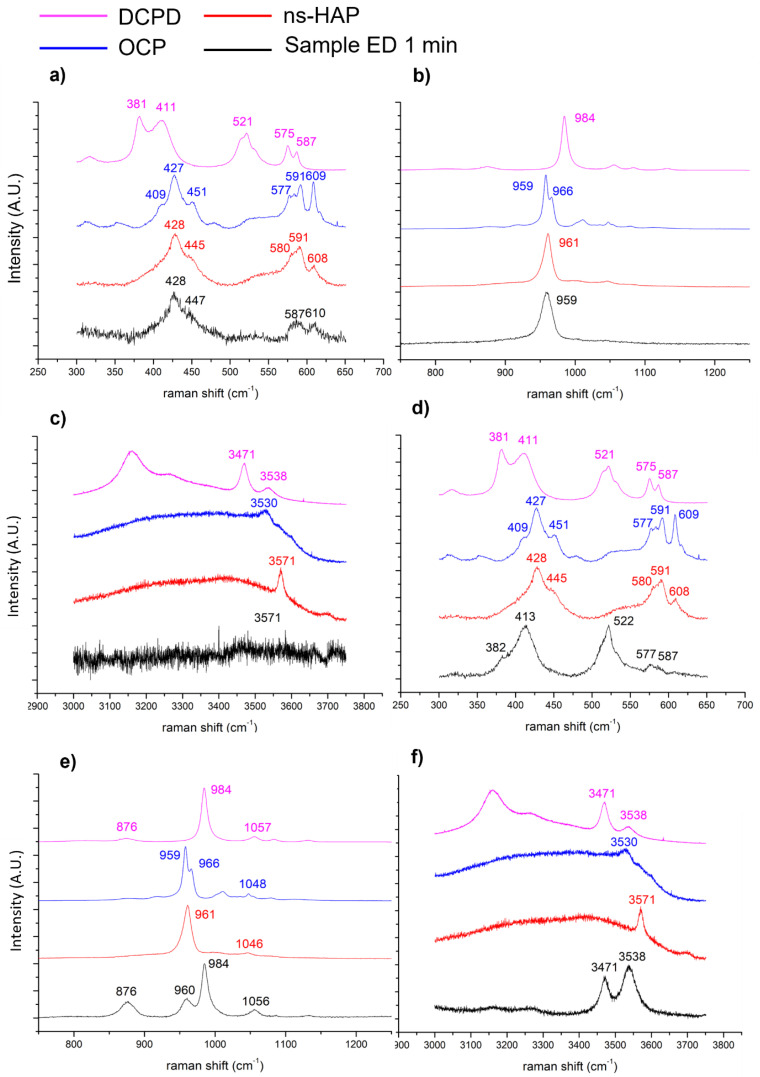
Raman spectra of DCPD, OCP, and ns-HAP reference powders and CaP-coated sample after 1 min of electrodeposition: (**a**–**c**) on the layer of nano-needles, and (**d**–**f**) on micro-platelet crystals.

The Raman spectrum recorded on the layer of nano-needles of the 1 min-coated sample (Figure 1b) shows an intense band at 959 cm^−1^ (Figure 3b) which could correspond to the ν_1_ PO_4_ vibration mode of the ns-HAP observed at 961 cm^−1^ on the reference compound spectrum. Nevertheless, this band is broad and dissymmetrical toward the higher wavenumbers, and then could also be attributed to poorly crystallized OCP whose widened bands at 959 and 966 cm^−1^ would have been superimposed into a single asymmetric one. The bands at 428, 447, 587, and 610 cm^−1^ (Figure 3a) correspond to the ν_2_ and ν_4_ vibration modes of PO_4_ of the ns-HAP and/or OCP. Indeed, these ones are poorly resolved and the associated bands to these vibration modes for the reference compounds of ns-HAP and OCP are very close. It has to be noted that the band corresponding to the OH-stretching of ns-HAP at 3571 cm^−1^ is not observable on the coated sample spectrum (Figure 3c). This may suggest that the layer of nano-needles is composed of nanocrystalline OCP. Moreover, a similar spectrum was obtained on the coating formed after 30 s of electrodeposition.

In addition, the spectrum obtained locally on the micrometric platelets observed on this coating shows an intense band at 984 cm^−1^ corresponding to the ν_1_ PO_4_ vibration mode of DCPD (Figure 3e). This is confirmed by the presence of the band at 876 cm^−1^ attributable to the P-OH bonds of HPO_4_^2−^ in DCPD, and the one at 1056 cm^−1^ attributable to the ν_3_ PO_4_ of this phase (Figure 3e). It is also possible to observe the bands at 382 and 413, and 522, 577, and 587 cm^−1^ (Figure 3d), corresponding to the ν_2_ and ν_4_ PO_4_ vibration modes of the DCPD, respectively. The bands at 3471 and 3538 cm^−1^ related to the O-H bonds of structural water in the DCPD phase are also visible (Figure 3f). Note that the band at 960 cm^−1^ (Figure 3e) comes from the layer of OCP nano-needles previously discussed, which is located under the DCPD crystals. The platelet morphology of the DCPD phase is characteristic, and, therefore, XRD analysis and Raman spectroscopy confirm its presence in the coating formed after 1 min of electrodeposition.

**Figure 4:**
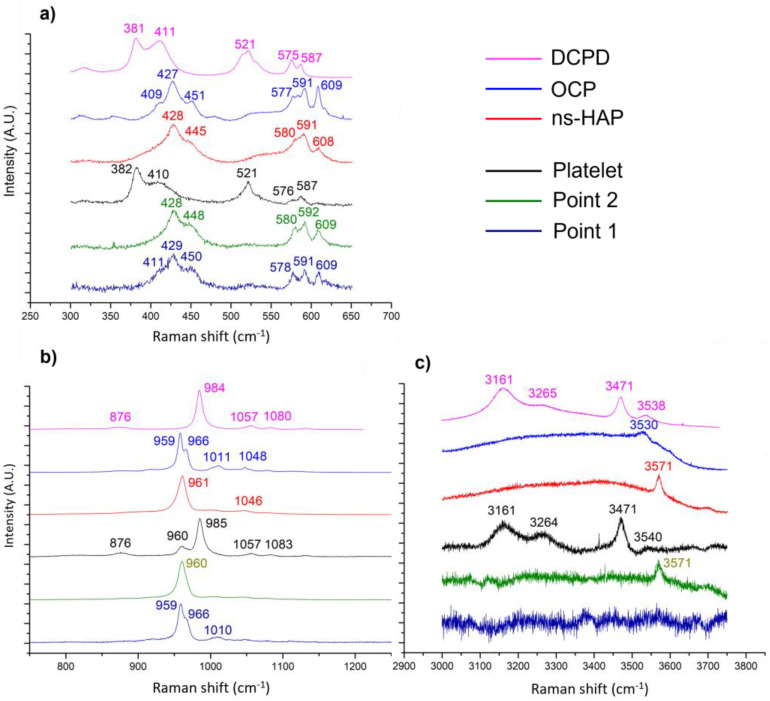

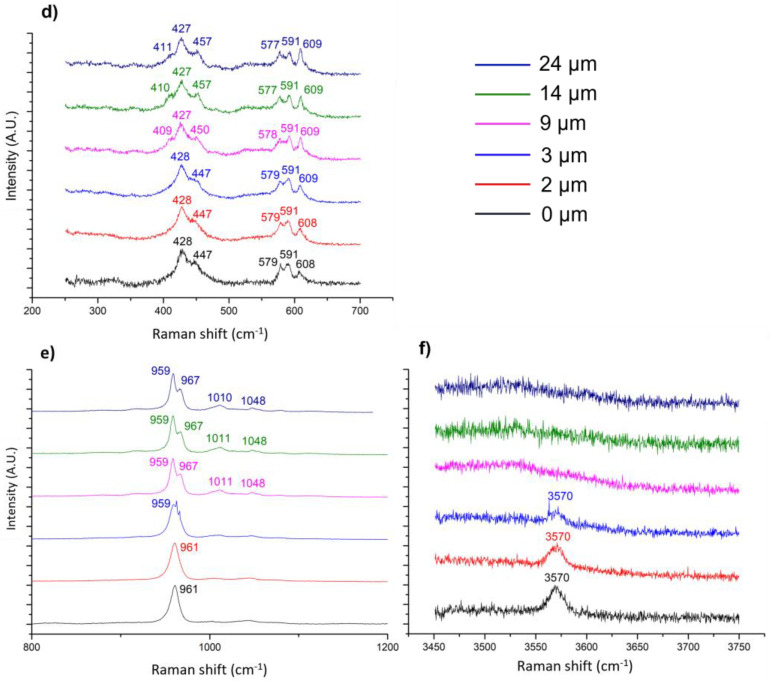
Raman spectra in three domains of interest: (**a**,**d**) 250–700 cm^−1^, (**b**,**e**) 800–1200 cm^−1^, (**c**) and (**f**) 3450–3750 cm^−1^) of (**a**–**c**) ns-HAP, OCP, DCPD reference powders, and three different morphologies (platelets, and points 1 and 2) observed for a CaP-coated sample after 10 min of electrodeposition, and (**d**–**f**) following the profile of a cross-section from the substrate surface (0 µm) to the top of the coating (24 µm).

Both morphologies observed on the coating obtained after 10 min of electrodeposition (Figure 1i–l) were also analyzed by Raman spectroscopy: two points on the layer of needles (named point 1 and point 2 in Figure 4a–c) and one on a micrometer platelet (named platelet in Figure 4a–c). The position of the bands (Figure 4a,b) corresponding to point 1 analysis confirmed that the needles layer is composed of OCP even more clearly than in the case of the 1 min coating because of the sharper bands due to enhanced crystallization. However, at another analyzed point (point 2) on the needles layer, a specific band at 3571 cm^−1^ (Figure 4c) corresponding to the OH-stretching vibration of the ns-HAP allows us to conclude that the phase is the ns-HAP instead of the OCP.

The Raman analysis of the platelets for this coating confirms that the associated phase is DCPD as already established for the coating deposited after 1 min.

A Raman microspectroscopy analysis profile was then recorded on the cross-section of the coating formed after 10 min as previously observed by SEM (Figure 1k,l) in order to study the distribution of the phases identified on the surface along the coating thickness. Figure 4d–f show the Raman spectra recorded from 0 μm (near the titanium substrate surface) up to 24 μm (the outer coating surface). It is possible to observe that the first three spectra (in the few 5 µm close to the substrate) have bands at 961 and 959 cm^−1^ and at 3570 cm^−1^ (Figure 4e,f) corresponding respectively to the ν_1_ PO_4_ vibration mode and to the OH-stretching of the ns-HAP. The three following spectra (above 10 µm thick) have the two bands at 959 and 967 cm^−1^ of the ν_1_ PO_4_ of OCP, and the band between 409 and 411 cm^−1^ (Figure 4d) associated with the ν_2_ PO_4_ of OCP (much less intense band for the ns- HAP (shoulder)). It should also be noted that there is no band at 3570 cm^−1^ (Figure 4f). It is therefore possible to conclude that the CaP phases composing the coating are distributed along the coating thickness: the layer closest to the substrate surface is composed of ns-HAP while the farthest one corresponds to OCP.

Calcium and phosphorus concentrations in the 1 min electrodeposited coating were determined by ICP spectrometry: the resulting Ca/P ratio is equal to 1.30 ± 0.01. This ratio is very close to the OCP one (Ca/P = 1.33); it confirms that the main constituent of the coating, appearing as the nano-needles layer observed for short electrodeposition duration, is OCP.

All in all, the 1 min electrodeposited CaP coating was selected for further investigation (copper-doping by ion exchange in solution) as the corresponding coating thickness meets the requirements (a few micrometers) and the coating, mainly composed of nano-needles of OCP, is homogeneously covering the implant surface.

### 3.2. Cu-Doping of the CaP Coating

**Figure 5:**
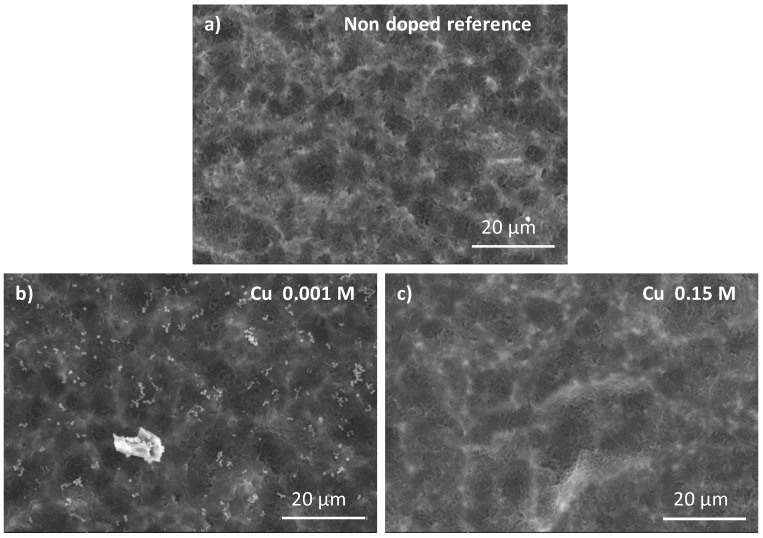
SEM micrographs of an undoped CaP-coated sample after 1 min of electrodeposition (**a**), after its doping by an ion exchange post-treatment using a copper ion solution of 0.001 M (**b**) or of 0.15 M (**c**).

The morphologies of Cu-doped CaP coatings and a non-doped coating (reference, CaP-coated sample obtained after 1 min of electrodeposition) are shown in Figure 5. Comparing to the reference (Figure 5a), the presence of nanoparticles on the sample surface doped using a solution with a copper concentration of 0.001 M (Figure 5b) are observed, whereas no change is detected for all the other concentrations up to 0.15 M (Figure 5c). EDX analysis indicates for all the samples the presence of calcium, phosphorus, and copper, demonstrating the association of Cu with the CaP coating after copper ion exchange post-treatment (see Figure S3 for a copper-doping with a 0.001 M solution).

**Figure 6:**
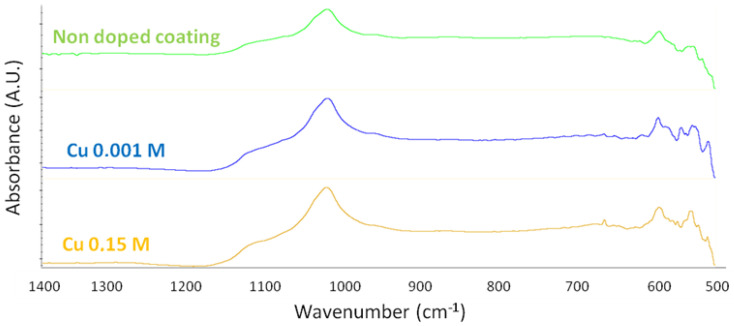
FTIR-ATR spectra of copper-doped CaP-coated samples using 0.001 M and 0.15 M copper solutions for ion exchange post-treatment in comparison with an undoped CaP-coated sample (obtained after 1 min of electrodeposition).

Then, no phase modification was identified by FTIR-ATR and Raman spectroscopy or XRD analyses compared to the reference sample (undoped), whatever the copper solution concentration used (Figure 6), despite the particles observed on the sample treated with a Cu 0.001 M solution (Figure 5b).

**Figure 7:**
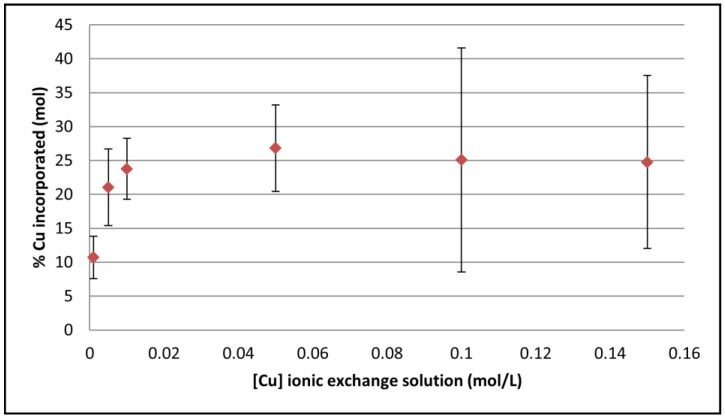
Molar percentage of copper ions incorporated in the coating versus total cations (Cu/(Cu+Ca) molar ratio in %) in relation to the concentration of the copper ion exchange solution.

The results of copper titration in the coating using AAS are presented in Figure 7 showing that a maximum Cu-doping level is reached using an ionic exchange post-treatment solution with a concentration equal or greater than 0.01 M. We can note that the relative quantity of copper incorporated in the CaP coating is quite high: the Cu/(Cu+Ca) molar ratio in the coating varied from 11 to 27 % depending on the copper concentration in the ionic exchange solution (Figure 7). The high standard deviations can be explained by the fact that the incorporation of antibacterial ions into a thin coating is a complex phenomenon. It depends on many factors not always controlled during the experiment i.e., the exposed surface which can vary from one sample to another, the temperature, the wettability of the coating, etc.

### 3.3. In Vitro Biological Properties

#### Antimicrobial Activity of Copper-Doped CaP Coating on Biofilm

**Figure 8:**
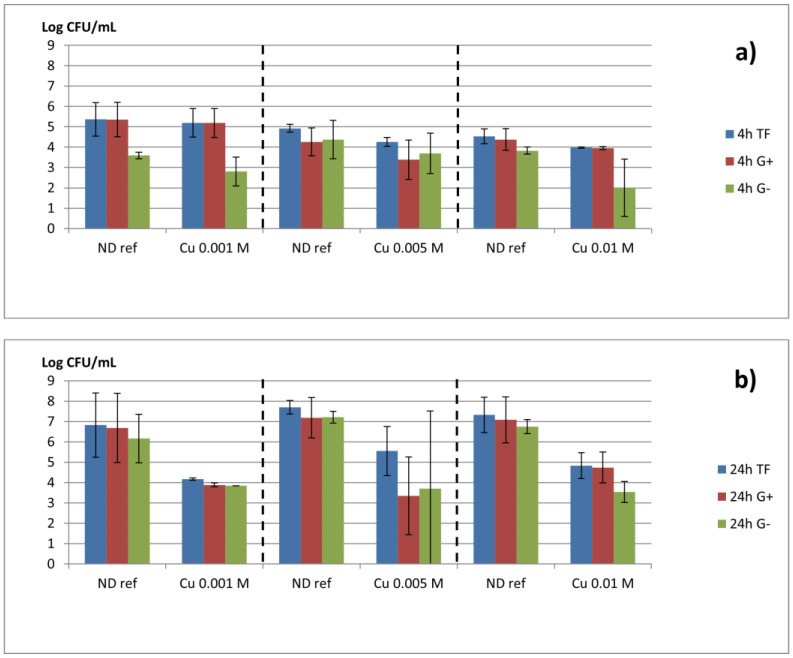
CFU/mL (mean ± SD) of Gram-positive (G+) and negative (G−) strains and total flora (TF) on non-doped CaP-coated samples (ND ref) and on Cu-doped CaP-coated samples (after exchange in 0.001, 0.005, or 0.01 M copper ion solutions) after 4 h (**a**) and 24 h (**b**) from inoculation.

Three different assay series were conducted depending on the copper doping (0.001 M, 0.005 M and 0.01 M). During each assay, the antibiofilm activity was assessed two times after inoculation. The numbers of adherent CFU 4 h after inoculation and incubation are presented in Figure 8a for total flora (TF), Gram-positive bacteria (G+), and Gram-negative bacteria (G−). For the non-doped CaP-coated samples (ND ref), the total adherent flora is higher than 4–5 log CFU/mL of recovery solution, and Gram-positive strains usually predominate after 4 h, confirming the involvement of primary colonizers. The G− population varies from 2 to 4 log CFU/mL. For all the samples doped with copper, after 4 h, a low reduction is noted in comparison to the corresponding ND ref especially for Gram-negative bacteria for the highest Cu load, whereas no noticeable difference is observed for Gram-positive bacteria and the total flora.

After 24 h from inoculation (Figure 8b), the consortium developed for the non-doped samples (ND ref) reaches about 7 to 8 log of adherent bacteria/mL, and the different species inoculated were recovered from the different culture media, demonstrating adhesion and growth of adherent bacteria with a mixed population combining G+ and G−. The results are highly reproducible between the different non-doped samples demonstrating the robustness of the model. For the samples doped with copper, after 24 h, a significant reduction of at least 2 log units for Gram-positive and Gram-negative strains as well as the total flora was observed. However, no noticeable dose-dependent effect was observed.

**Figure 9:**
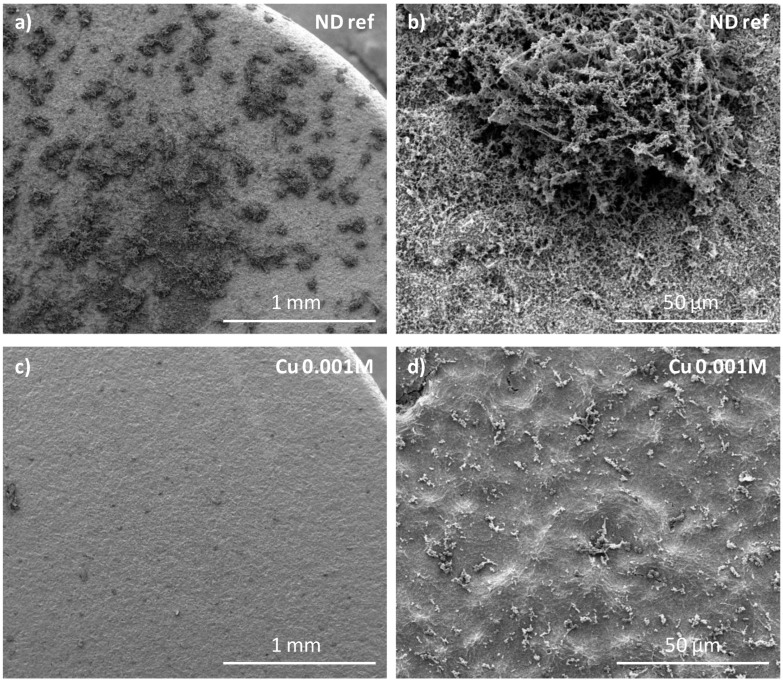
SEM micrographs of (**a**,**b**) non-doped CaP-coated sample (ND ref) and (**c**,**d**) copper-doped CaP-coated sample with 0.001 M copper ion solution after 24 h of immersion in biofilm model broth.

These results (Figure 8) are confirmed by SEM observations (Figure 9) showing micrographs of a non-doped CaP-coated sample and a copper-doped CaP-coated sample (Cu 0.001 M) after 24 h of biofilm formation. Much less bacteria can be observed on the copper-doped sample (Figure 9c,d) coherent with the reduction of more than 2 log units observed by CFU counts (Figure 8b).

Complementarily, we tested the cytotoxicity of ND ref CaP-coated and Cu-doped CaP-coated samples by direct contact with mammalian cells (Vero cell layer) according to the EN/ISO 10993-5 standard (see in the Supplementary Materials, n°4-Cytotoxicity on mammalian cells). The results showed no effect on viability (Figure S4) even for a sample with one of the higher copper incorporation rates in the CaP coating (Cu/(Cu+Ca) molar ratio = 25%, obtained with the 0.01 M copper ion exchange solution, Figure 7) for which the viability is higher than 77%.

## 4. Discussion

### 4.1. On the Formation of a Calcium-Phosphate-Electrodeposited Coating on Titanium Substrate

The formation of the CaP phases on the titanium substrates can be explained by the electrochemical reactions. Indeed, when the potential is applied between the electrodes, redox reactions occur at each electrode surface. Water, which is the solvent of the electrolyte solution, is mainly involved according to the following reactions (Equations (1) and (2)) [47,53,54]:

Oxidation:

(1) $$2{\;H}_{2}O\;\to {\;O}_{2}↑+4{\;H}^{+}+4{\;e}^{-}$$

Reduction:

(2) $$2{\;H}_{2}O\;+2e^{-}\;\to {\;H}_{2}↑+2{\;\mathrm{OH}}^{-}$$

Moreover, in this study, the pH of the electrolyte being slightly acidic (pH = 4–4.5), proton reduction can also occur as described in the literature [38,55]:

(3) $$2{\;H}^{+}+2e^{-}\;\to {\;H}_{2}↑$$

The titanium sample being the cathode of the electrodeposition system, it is water reduction that is the most important reaction, inducing the creation of OH^−^ ions at the implant surface, leading to a local pH increase. This pH change then induces acido-basic reactions. These result in the dissociation of dihydrogenphosphate ions contained in the electrolyte, as follows (Equations (4) and (5)) [46,47,54]:

(4) $$H_{2}{\mathrm{PO}}_{4}^{-}+{\;\mathrm{OH}}^{-}\;\to {\;\mathrm{HPO}}_{4}^{2-}+{\;H}_{2}O\;$$

(5) $${\mathrm{HPO}}_{4}^{2-}+{\;\mathrm{OH}}^{-}\;\to {\;\mathrm{PO}}_{4}^{3-}+{\;H}_{2}O$$

Finally, the previously described acido-basic reactions lead to calcium and phosphate supersaturation at the surface of the titanium substrate, inducing precipitation of the less soluble CaP phase(s) at 60 °C depending on the pH. In accordance with pH value and the phosphate ions that compose the electrolyte, several CaP phases can precipitate [38,46,47]:

1. Brushite (DCPD):

(6) $${\mathrm{Ca}}^{2+}+{\mathrm{HPO}}_{4}^{2-}+2{\;H}_{2}O\;\to {\;\mathrm{CaHPO}}_{4}.2H_{2}O\;$$

2. Octacalcium phosphate (OCP):

(7) $$8{\;\mathrm{Ca}}^{2+}+2{\;\mathrm{HPO}}_{4}^{2-}+4{\;\mathrm{PO}}_{4}^{3-}+5{\;H}_{2}O\;\to {\;\mathrm{Ca}}_{8}{\left({\mathrm{HPO}}_{4}\right)}_{2}{\left({\mathrm{PO}}_{4}\right)}_{4}.5H_{2}O$$

3. Non-stœchiometric apatite (ns-HAP):

(8) $$\left(10-x\right){\;\mathrm{Ca}}^{2+}+{x\;\mathrm{HPO}}_{4}^{2-}+\left(6-x\right){\;\mathrm{PO}}_{4}^{3-}+\left(2-x\right){\;\mathrm{OH}}^{-}\;\;\to {\;\mathrm{Ca}}_{10-x}{\left({\mathrm{HPO}}_{4}\right)}_{x}{\left({\mathrm{PO}}_{4}\right)}_{6-x}{\mathrm{OH}}_{2-x}\;\;\;\;\;\;\;\;\;\;\;\;\;\mathrm{with}\;0\;<\;x\;<\;2$$

4. Stoichiometric hydroxyapatite (HAP):

(9) $$10{\;\mathrm{Ca}}^{2+}+6{\;\mathrm{PO}}_{4}^{3-}+2{\;\mathrm{OH}}^{-}\;\to {\;\mathrm{Ca}}_{10}{\left({\mathrm{PO}}_{4}\right)}_{6}{\mathrm{OH}}_{2}$$

It was observed that the electrodeposition time has a significant effect on the coating morphology and phases content. Indeed, for a short electrodeposition time, a layer of nano-needles is observed, whereas for a longer electrodeposition time, two layers are formed: one composed of previously observed nano-needles, and the other one of micrometric ones. This growth mechanism of the coating is in agreement with the one presented by Mokabber et al., describing time-dependent growth of CaP crystals [55]. They explained that nucleation rate and crystal morphology are mainly affected by the degree of supersaturation. In this case, pH can be considered as the main driver of supersaturation, and depends on the OH^−^ ions created by the electrochemical reactions, and, therefore, on the distance to the sample surface as well as their consumption during the formation of the CaP crystals. During the first stage of the deposition, the electrolyte is highly supersaturated leading to randomly oriented polycrystals, because the structural match between the newly formed nuclei and the substrate becomes less important [56]. Later, the crystal growth occurs with a lower supersaturation because of a larger distance from the sample surface. Moreover, the crystal growth is hindered in the direction parallel to the surface due to the other crystals and can more easily develop perpendicularly to the surface. This prevents a randomly oriented nucleation and growth leading to crystals with a preferred orientation as observed in Figure 1k,j.

The different CaP phases observed for short and long deposition times could be explained by different pH, leading to various predominant phosphate ions (i.e., H_2_PO_4_^−^, HPO_4_^2−^, PO_4_^3−^) that can react with calcium to form DCPD, OCP, or ns-HAP. Nevertheless, the local pH is extremely difficult to measure to confirm this hypothesis. Moreover, the presence of these ions does not allow an obvious explanation of the CaP phases that precipitate. Indeed, Eliaz and Sridhar report that the CaP system is very complex and that thermodynamic factors like the solubility product are not sufficient to predict the first phase that precipitates [57]. Another factor is the dichotomy between the thermodynamic stability of the mineral phases and their respective rates of nucleation and crystal growth. For example, an amorphous or crystalline phase can precipitate and then undergo a rapid transformation to a more stable crystalline phase. That is why a kinetic aspect has to be taken into account, and is described by the Ostwald’s step rule: during the phase formation (crystallization, fusion, or condensation), the first phase that nucleates is not the most thermodynamically stable one, but the one that has the free energy closest to the original state [38]. In accordance with this law, amorphous calcium phosphate (ACP), DCPD, and OCP are considered as apatite precursor phases in vivo, in vitro, and during electrodeposition because their formation rates are faster than the one of ns-HAP [58,59]. Based on calculations of free enthalpy (ΔG) and nucleation rate (J), Wang et al. showed that for electrodeposition, HAP is thermodynamically more stable than OCP which is itself more stable than DCPD (ΔG(HAP) > ΔG(OCP) > ΔG(DCPD) in absolute value) for a pH greater than 6. On the other hand, the nucleation rate of DCPD is always higher than that of OCP which is itself always superior to that of HAP (J(DCPD) > J(OCP) > J(HAP)) [60]. Eliaz and Sridhar showed that, for a solution at 60 °C, the DCPD should not precipitate, whereas HAP can be formed for a pH greater than 7.2, and OCP can precipitate in any pH range [57]. It should be noted that few studies are conducted on ns-HAP. Nonetheless, Lu and Leng reported that the precipitation of ns-HAP is less favorable thermodynamically than HAP and is similar to OCP, and that the nucleation rate is higher than that of stoichiometric HAP and therefore closer to the OCP one [61]. Finally, the transformation of OCP into HAP can occur via: (1) a process of OCP dissolution and reprecipitation of HAP crystals, and (2) in situ hydrolysis (by topotactic transformation) accompanied by the calcium consumption of the surrounding solution and/or release of phosphate ions in the solution [62]. These previous studies allow us a better understanding of the CaP formed during the present work: OCP and DCPD for a short deposition time, and DCPD, OCP, and ns-HAP for a longer deposition time. It has also been highlighted that for those long electrodeposition times, the coating closest to the substrate is composed of ns-HAP whereas the farthest is composed of OCP. For the short deposition time, OCP precipitation can be explained by the fact that this is kinetically favored even if it is thermodynamically less stable than apatite. For the longer deposition time, the OCP crystals that precipitated during the first stage of electrodeposition on the substrate have time to hydrolyze into apatite, according to Equation (10) or Equation (11) [58]. Note that these reactions are written to form HAP but can be adapted to lead to ns-HAP. In the case of this study, the reaction (10) seems more favorable because calcium ions are available in the electrolyte during the reaction.

(10) $${\mathrm{Ca}}_{8}H_{2}{\left({\mathrm{PO}}_{4}\right)}_{6}\cdot 5H_{2}O\;+\;2\;{\mathrm{Ca}}^{2+}+\;4\;H_{2}O\;\to \;2\;{\mathrm{Ca}}_{5}{\left({\mathrm{PO}}_{4}\right)}_{3}\mathrm{OH}\;+\;3\;H_{2}O\;+\;4\;H_{3}O^{+}$$

(11) $$1.25\;{\mathrm{Ca}}_{8}H_{2}{\left({\mathrm{PO}}_{4}\right)}_{6}\cdot 5H_{2}O\to \;2\;{\mathrm{Ca}}_{5}{\left({\mathrm{PO}}_{4}\right)}_{3}\mathrm{OH}\;+\;1.5\;H_{3}{\mathrm{PO}}_{4}\;+\;4.25\;H_{2}O_{}$$

Then, the newly formed crystals, meaning farthest from the cathode surface, do not have time to hydrolyze and will remain OCP. The presence of this phase can also be explained by its distance from the titanium substrate surface, leading to a lower pH that can promote its formation and hinder its hydrolysis into apatite.

In all cases, the precipitation of some large DCPD crystals, as observed above the coating, can come from the distance from the cathode surface, and therefore from a more acidic pH, closer to the electrolyte pH (equal to 4.5). In addition, OCP precipitation is governed by Equations (12) and (13) [62]. It should be noted that the reaction (13) seems most appropriate in our synthetic conditions because it involves HPO_4_^2−^ ions and not PO_4_^3−^ ones, which are certainly not in the majority in the electrolyte. In addition, according to this equation, OCP precipitation results in partial acidification (H_3_O^+^ ions release) and therefore promotes the formation of DCPD once again. Furthermore, the previous Equation (10) can also generate local acidification.

(12) $$8{\;\mathrm{Ca}}^{2+}+2{\;\mathrm{HPO}}_{4}^{2-}+4{\;\mathrm{PO}}_{4}^{3-}+5{\;H}_{2}O\;\to {\;\mathrm{Ca}}_{8}{\left({\mathrm{HPO}}_{4}\right)}_{2}{\left({\mathrm{PO}}_{4}\right)}_{4}\cdot 5H_{2}O\;$$

(13) $$8{\;\mathrm{Ca}}^{2+}+6{\;\mathrm{HPO}}_{4}^{2-}+9{\;H}_{2}O\;\to {\;\mathrm{Ca}}_{8}{\left({\mathrm{HPO}}_{4}\right)}_{2}{\left({\mathrm{PO}}_{4}\right)}_{4}\cdot 5H_{2}O+4\;H_{3}O^{+}$$

To summarize, different CaP phases can be formed by electrodeposition on pure titanium substrate and it is dictated by the deposition time. In all cases, the CaP coatings should favor implant osseointegration. As already indicated, an electrodeposition time of 1 min was chosen for the antibacterial doping.

**Figure 10:**
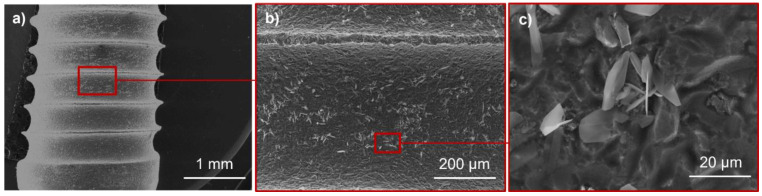
SEM micrographs showing an undoped CaP-coated titanium dental implant after 1 min of electrodeposition at three magnifications (from low (**a**) to higher magnification (**b**) and then (**c**). The focus area corresponding to (**b**,**c**) is indicated by the red rectangle on (**a**,**b**), respectively).

The experiments and associated results presented were performed on titanium cylindrical coupons to facilitate the physico-chemical characterization of the substrate surface and CaP coating. In addition, a study on real implants was conducted to verify that the same coating morphology and associated phases were obtained. Figure 10 shows the CaP coating formed on a titanium dental implant surface, similar to what was obtained on the simplified geometry samples.

**Figure 11:**
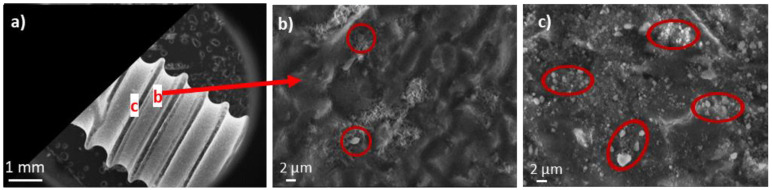
SEM micrographs of an undoped 1 min-CaP-coated titanium dental implant after the screwing and unscrewing test in an artificial jawbone: (**a**) General view and indication of the location of b and c focus area; (**b**) At the thread bottom; and (**c**) At the thread top of the dental implant. The red circles show that some bright particles corresponding to debris of the artificial jawbone remained stuck to the implant.

In addition, the mechanical stability of this CaP coating on the dental implant surface was evaluated by SEM after the screwing and unscrewing test in an artificial jawbone (Figure 11). No alteration of the CaP coating was noticed at the thread bottom of the coated implant (Figure 11b). Some bright particles corresponding to debris of the artificial jawbone remained stuck to the implant (red circles in Figure 11b) and may result from warming at the implant/artificial jawbone interface during the screwing and unscrewing processing, leading to a partial melting of the plastic. On the thread top (Figure 11c), much more particles of resin from the artificial jawbone are visible. Nevertheless, we can see the coating below and next to these resin particles (Figure 11b,c).

### 4.2. Cu-Doped Calcium Phosphate Coating and Their Related Microbiological Properties

Instead of adding copper directly in the electrolyte as usually described in the literature [40,63] and to prevent the formation of metallic particles in the coating, the antibacterial agent (Cu^2+^ ions) was incorporated as ions into the coating by an ionic exchange post-treatment of the CaP-coated samples following Equation (14). Such a process has already been described for the exchange of magnesium, strontium, or zinc with the calcium ions composing a CaP coating [64,65,66]. Copper ion is of interest because it demonstrates a high antibacterial activity [41,63], its ionic charge is similar to that of calcium ion (+2), and its ionic radius is not too far from the Ca one (73 pm for Cu(II) and 100 pm for Ca [67]) that could allow a high exchange rate.

(14) $${\mathrm{Cu}}_{\mathrm{solution}}^{2+}+{\;\mathrm{Ca}}_{\mathrm{solid}}^{2+}\;↔{\;\mathrm{Cu}}_{\mathrm{solid}}^{2+}+{\;\mathrm{Ca}}_{\mathrm{solution}}^{2+}$$

It has been shown that the incorporation of Cu^2+^ in the CaP coating does not affect its phases content. Regarding the quantity of copper incorporated, it reaches a plateau for a Cu^2+^ concentration in the exchange bath greater or equal to 0.01 M. This phenomenon has been already observed by Drouet et al. who highlighted a Langmuir adsorption isotherm in the case of the ion exchange of divalent cations such as Mg^2+^ and Sr^2+^ with Ca^2+^ for nanocrystalline non-stoichiometric apatite [66]. It indicates that the amount of exchangeable ions in the case of copper is limited because only the surface reacts with the exchange solution and is finally saturated. It is also possible to note that the quantity of incorporated copper is high (11 to 27%), resulting from the fact that calcium and copper ions have quite the same characteristics, such as their charge and their ionic radii [67].

Recent publications underlined the antimicrobial properties of an implant coated with copper, referring to the inhibition of the growth (agar diffusion method) of aerobic bacteria. To our knowledge, this is the first report with a demonstration of a specific antibiofilm activity with respect to the microbial ecology described in peri-implantitis. In our conditions, the non-doped samples (ND ref) are characterized by adhesion (4 h) then colonization (24 h) by a mixed biofilm formed by all the species inoculated and with a balance Gram+ Gram– which can reach 7 to 8 log. These observations concerned all the assays performed with ND ref (2 × 3) demonstrating the robustness of our model. Finally, copper-doped CaP coatings have shown an antibiofilm effect against the bacterial strains encountered in peri-implantitis for primary to secondary colonizers. The plateau for the doping rate being reached at 0.01 M, samples doped with concentrations of copper lower or equal to 0.01 M were tested. In the described assay conditions, no noticeable effect was observed 4 h after inoculation which can indicate a limited activity on early bacterial adhesion. In opposition, a significant difference was noted after 24 h of incubation on the proliferation of adherent bacteria for the three tested concentrations (i.e., 0.001 M, 0.005 M, 0.01 M). No dose effect was observed, indicating that 11% of copper incorporation in the coating is sufficient to impair the implant colonization, and a two-fold increase in Cu incorporation does not improve this effect. Another copper dose range leading to lower Cu incorporation in the CaP coating should be tested in future work in view of determining the lower copper rate that is effective on the biofilm model. Finally, for the three tested concentrations, no significant decrease in mammalian cell viability was noted (Figure S4), even for the higher copper incorporation rate in the CaP coating. These results are in favor of complementary in vivo assays with regard to biocompatibility before use.

## 5. Conclusions

The purpose of this study was to develop a homogeneous and adherent thin (a few micrometers) CaP antibacterial coating on titanium dental implants by electrodeposition in view of favoring implant osseointegration and limiting the postoperative peri-implantitis risks. After 1 min of electrodeposition with a constant potential of −1.6 V/SCE at 60 °C, we obtained a 2 μm thick CaP coating on titanium substrates (titanium coupons and titanium dental implants). As characterizing such a thin coating on a rough titanium substrate (1.4 < Ra < 1.6 µm) is challenging, we had to combine a multitool approach involving global (XRD, FTIR) and local (Raman spectroscopy on the coating surface and along the cross-section, SEM) analysis techniques to succeed in determining the coating phases content as a function of the electrodeposition time. This study showed that the microstructure of the electrodeposited CaP coatings formed during electrodeposition on titanium substrate depends on electrolyte supersaturation, related to the local pH, and then on the distance between the crystals formed and the implant surface at the cathode, whereas the chemical composition depends also on the local pH and on the kinetic reaction. Indeed, CaP precipitation during electrodeposition seems to be controlled more by a kinetic step than a thermodynamic one. To avoid the possibility of copper metallic particles forming in the electrodeposited coating, we implemented a strategy without introducing copper ions in the electrolyte solution, which is generally used, but instead, more preferably, by an ion exchange post-treatment. We showed that quite high incorporation rates of copper in the CaP coating can be reached (up to 27% for the Cu/(Cu+Ca) molar ratio) with a copper concentration in the ionic exchange solution ≥ 0.01 M (but this is limited due to surface saturation), without affecting CaP phases content in the coating. To the best of our knowledge, this is the first study demonstrating a specific antibiofilm activity with respect to the microbial ecology described in peri-implantitis. All the assays performed with the non-doped samples demonstrate the robustness of the multispecies biofilm model we implemented. Finally, copper-doped CaP coatings have shown an antibiofilm effect against the bacterial strains encountered in peri-implantitis for primary to secondary colonizers without a noticeable dose effect, indicating that the lower copper incorporation rate (11%) in the CaP coating obtained in our conditions is sufficient to impair the implant colonization without being cytotoxic. This study demonstrates that combining the electrodeposition and ionic exchange processes appears as a promising route to producing antibacterial CaP-coated titanium dental implants with tunable thickness and copper doping.

## Figures and Tables

**Table 1:** List of bacterial strains, incubation conditions, and culture media used.

	Tested Strains	Reference	Subculturing Media	Incubation Conditions
Gram-positive bacteria	*Streptococcus gordonii*	CIP 105258T	Columbia agar + 5% sheep blood	36 °C, anaerobiosis
	*Actinomyces naeslundii*	CIP 103128T	Columbia agar + 5% sheep blood	36 °C, anaerobiosis
	*Parvimonas micra*	CIP 105294T	Columbia agar + 5% sheep blood	36 °C, anaerobiosis
Gram-negative bacteria	*Fusobacterium nucleatum*	CIP 101130T	Columbia agar + 5% sheep blood	36 °C, anaerobiosis
	*Aggregatibacter actinomycetemcomitans*	CIP 52106T	Columbia agar + 5% sheep blood	36 °C, 5% CO_2_
	*Prevotella intermedia*	CIP 103607	Columbia agar + 5% sheep blood	36 °C, anaerobiosis
	*Porphyromonas gingivalis*	CIP 103683	Wilkins Chalgren + 5% horse blood	36 °C, anaerobiosis

**Table 2:** Thickness measurements (in µm) and their respective standard deviations of calcium phosphate coatings electrodeposited during 30 s, 1, 2, 5, and 10 min, by SEM cross-sectional observation, calotest, and AFM techniques.

	Technique	SEM	Calotest	AFM
Time (min)				
0.5		1.6 ± 0.2	-	0.97 ± 0.04
1		2 ± 1	1.9 ± 0.2	1.07 ± 0.04
2		2.7 ± 0.5	-	2.15 ± 0.06
5		9 ± 2	6.0 ± 0.5	>3.6
10		13 ± 3	7.4 ± 0.7	>3.6

## Data Availability

Data are contained within the article and the associated Supplementary Materials file. Most of the data presented in this study are also available in the PhD manuscript of Camille PIERRE accessible at the following address: https://www.theses.fr/en/2018INPT0084 (accessed on 22 December 2022).

## Supplementary Materials

The following supporting information can be downloaded at: https://www.mdpi.com/article/10.3390/jfb14010020/s1, Figure S1: Experimental set for CaP electrodeposition on dental implant: a) General overview, and b) Focus on the dental implant placed at the cathode and the reaction of reduction of water occurring at the surface; Figure S2: FTIR-ATR spectra of DCPD, OCP, ns-HAP reference compounds and CaP coated samples after 1 and 10 min of electrodeposition: a) Full spectrum (4000–400 cm^−1^), and b) 1300–450 cm^−1^ domain; Figure S3: SEM micrograph (a) of a 1 min-electrodeposited CaP coating doped with an ion exchange post-treatment using a copper ion solution of 0.001 M, its corresponding EDX spectrum (b), and elemental analysis report (c); Methodology for Cytotoxicity assessment on mammalian cells: 4-Cytotoxicity on mammalian cells and Figure S4: Percentage of viability of Vero cells after direct contact with non-doped CaP coated samples (ND ref) and Cu-doped CaP coated samples after exchange in 0.001, 0.005 or 0.01 M copper ions solutions.
