# Supplementary material for: Antibacterial Electrodeposited Copper-Doped Calcium Phosphate Coatings for Dental Implants
Source: J Funct Biomater. 2022 Dec 29;14(1):20. doi: 10.3390/jfb14010020 (PMC9863956; doi:10.3390/jfb14010020)
Supplement: Supplementary file 1 [file jfb-14-00020-s001.zip › jfb-2106114-supplementary.pdf]

Supplementary material

Antibacterial electrodeposited copper-doped calcium phosphate coatings for dental implants

Camille Pierre[1], Ghislaine Bertrand[1], Iltaf Pavy[2], Olivier Benhamou[3], Christian Rey[1], Christine Roques[2], Christèle Combes[1]

[1]CIRIMAT, Université de Toulouse, CNRS, Toulouse INP - ENSIACET, Toulouse 31030, France

[2]LGC, Université de Toulouse, CNRS, Université Paul Sabatier, Faculté des Sciences Pharmaceutiques, Toulouse 31062, France

[3] Arts Loi Dental Clinic, Rue de la Loi 28, Bruxelles 1040, Belgium

1 – CaP coating on dental implant by electrodeposition

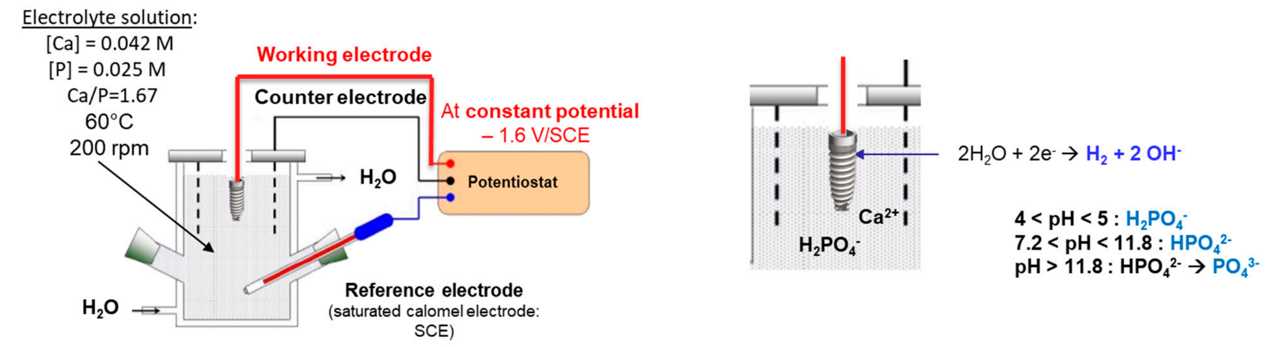

Figure S1: Experimental set for CaP electrodeposition on dental implant: a) General overview and, b) Focus on the dental implant placed at the cathode and the reaction of reduction of water occurring at the surface.

2 - FTIR-ATR analysis of CaP reference compounds and CaP coated samples

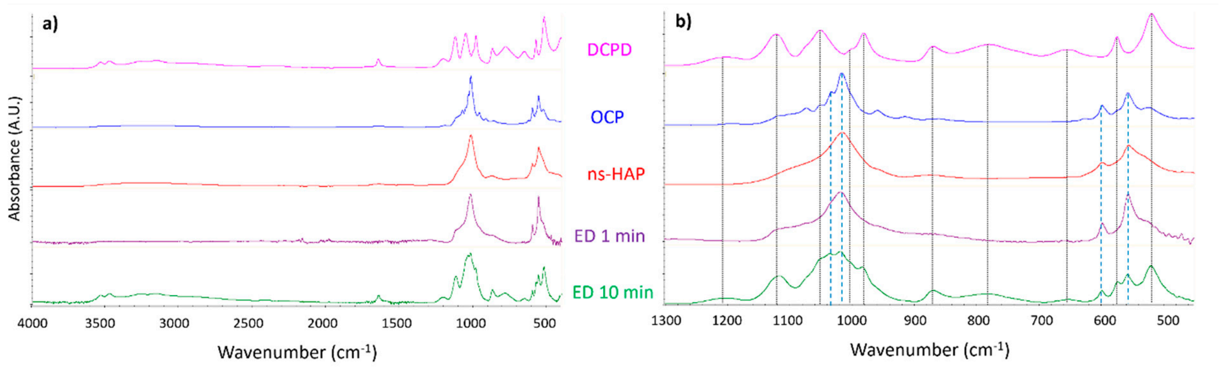

Figure S2: FTIR-ATR spectra of DCPD, OCP, ns-HAP reference compounds and CaP coated samples after 1 and 10 minutes of electrodeposition : a) Full spectrum (4000-400 cm^{-1}) and b) 1300-450 cm^{-1} domain.

3 – SEM-EDX analysis of Cu-doped CaP coated titanium

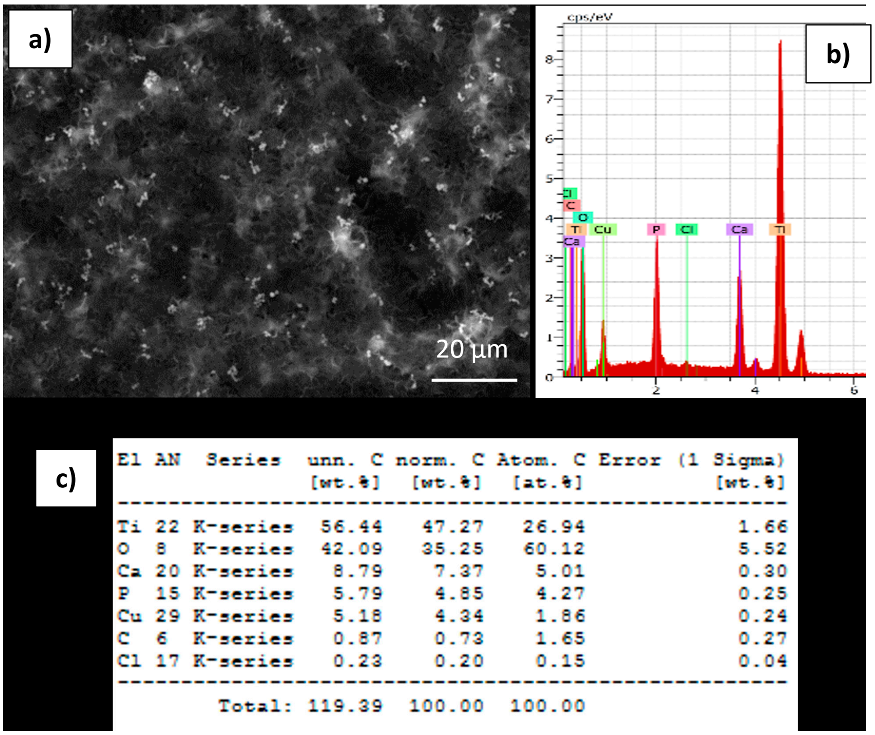

c)

El	AN	Series	unn. C [wt.%]	norm. C [wt.%]	Atom. C [at.%]	Error (1 Sigma) [wt.%]
Ti	22	K-series	56.44	47.27	26.94	1.66
O	8	K-series	42.09	35.25	60.12	5.52
Ca	20	K-series	8.79	7.37	5.01	0.30
P	15	K-series	5.79	4.85	4.27	0.25
Cu	29	K-series	5.18	4.34	1.86	0.24
C	6	K-series	0.87	0.73	1.65	0.27
Cl	17	K-series	0.23	0.20	0.15	0.04
		Total:	119.39	100.00	100.00	

Figure S3: SEM micrograph (a) of a 1 min electrodeposited CaP coating doped with an ion exchange post-treatment using a copper ion solution of 0.001 M, its corresponding EDX spectrum (b) and elemental analysis report (c).

4 - Cytotoxicity on mammalian cells

Evaluation of CaP coated titanium coupons cytotoxicity was performed using the MTT (3-(4,5-dimethylthiazol-2-yl)-2,5-diphenyltetrazolium bromide) colorimetric assay based on Mosmann *et al.* procedure [1] , according to the EN/ISO 10993-5 standard (tests for *in vitro* cytotoxicity) for direct contact test. The potential cytotoxic activity of the copper doped CaP-coated coupons (after ionic exchange in 0.005, 0.001 and 0.01 M copper nitrate solution) versus non-doped CaP-coated coupons was assessed against Vero cells (ATCC® CCL-81) purchased from ATCC® (Manassas, United-States) and cultivated in RPMI medium (RPMI 1640 – PAN-Biotech). 96-wells microtiter plates were first filled with 100 µL of a cell suspension prepared in RPMI medium (2 x 10^4 cells/100 µL). After overnight incubation at 37 °C in a humidified 5-6.5% CO_2 incubator, the cell confluence was checked by microscopic observation. Then, the medium was replaced by fresh medium and the coupons were placed gently on the cell layer in the centre of the well. Wells corresponding to the negative control were supplemented

with 100 µL of fresh medium. Tween 40 (5%, Sigma, St. Quentin Fallavier, France) was used as positive control for cytotoxicity. The microplate was then incubated for 24 h at 37 °C in a humidified 5-6.5% CO_2 incubator. The supernatant was then discarded followed by a rinsing with 100 µL of phosphate buffer solution (PBS). Then 100 µL of a MTT solution prepared in PBS at a concentration of 0.5 mg/mL were added in all wells. After 60 min of incubation at 37 °C and in order to solubilize the formed formazan, indicator of cell viability, 100 µL of DMSO were added. After agitation, the optical density (OD) was measured at 570 nm using a CLARIOstar Plus plate reader (BMG Labtech). Finally, viability percentages were determined by measuring the OD at 570 nm for each Cu-doped CaP-coated coupons, in comparison to the non-doped ones. Assays were performed in triplicate during a single test. The results are presented in Figure S3.

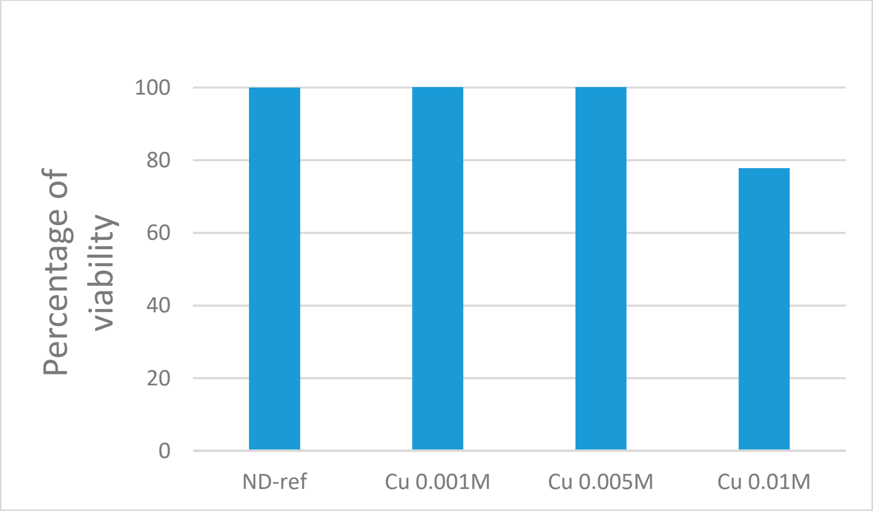

Figure S4: Percentage of viability of Vero cells after direct contact with non-doped CaP coated samples (ND ref) and Cu-doped CaP coated samples after exchange in 0.001, 0.005 or 0.01 M copper ions solutions.

Reference:

[1] Mosmann, T. Rapid colorimetric assay for cellular growth and survival: Application to proliferation and cytotoxicity assays. *Journal of Immunological Methods* **1983**, 65, 55-63, D.O.I.: https://doi.org/10.1016/0022-1759(83)90303-4.
